# Perspectives Toward Kidney Transplantation Amongst Individuals With Kidney Failure in Canada: A Qualitative Study

**DOI:** 10.1177/20543581261472120

**Published:** 2026-08-12

**Authors:** Cynthia Kendell, Jad Issa, Bethany Foster, Karthik Tennankore, Amanda J. Vinson

**Affiliations:** 1 Department of Medicine, 3688 Dalhousie University, Halifax, NS, Canada; 2 Department of Medicine, 432234 Nova Scotia Health, Halifax, NS, Canada; 3 Kidney Research Institute of Nova Scotia, Halifax, NS, Canada; 4 507266 Research Institute of the McGill University Health Centre, Montreal, QC, Canada

**Keywords:** kidney failure, kidney transplantation, patient perspectives, qualitative research

## Abstract

**Introduction:**

An understanding of perspectives toward kidney transplant amongst individuals with kidney failure is integral to supporting treatment-related decision-making and preparing for life post-transplant, yet this has not been studied in a Canadian context. Therefore, the objective of this study was to examine the perspectives of individuals with kidney failure in Canada regarding kidney transplant.

**Methods:**

Adults (18-75y) from four Canadian provinces who either had initiated dialysis within the prior 6 months or were preemptively referred for kidney transplantation within the prior 3 months were recruited to participate in focus group discussions (FGDs) or individual interviews. Data were analyzed using inductive thematic analysis.

**Results:**

Fifteen individuals participated in one of four FGDs (n=13) or a semi-structured interview (n=2). Seven were on dialysis and eight were pre-dialysis (four and six of whom had been referred for transplant, respectively). Overall, participants expressed a clear preference for transplant compared to dialysis. Participants’ willingness to consider transplant was influenced by their (1) *relationship with the healthcare team*, recognition of (2) *the necessity of treatment*, the (3) *potential to live a “normal” life*, and knowledge of (4) *others’ experiences with transplant*. Despite a preference for transplant, participants expressed (5) *concerns regarding securing a living donor* and a sense of having (6) *a lack of control* over the transplant referral process.

**Conclusion:**

In this study, we evaluated perceptions towards kidney transplantation amongst individuals with kidney failure in Canada. Understanding potential concerns regarding future transplant may lead to improved patient-centered education, communication and psychosocial support strategies to empower patients to confidently make treatment-related decisions.

## Introduction

Kidney transplantation is the best treatment option for patients with kidney failure, leading to improved survival and quality of life compared to dialysis.^1^ Despite these benefits, the process of receiving and living with a kidney transplant is associated with various stressors and the decision to pursue transplant is not always straightforward. Patient-expressed concerns include challenges with adherence to immunosuppressive medication, maintenance of a healthy lifestyle, and emotional apprehensions such as increased worry, anxiety, or feelings of guilt post-transplant.^2,3^ Compared with recipients of other solid organ transplants, kidney transplant recipients have been shown to worry significantly more about, and experience more feelings of guilt relating to their transplant.^4^

Understanding patient perspectives regarding kidney transplant is integral to supporting patients in shared treatment-related decision-making and preparing patients for life post-transplant. Currently, the literature on how and why potentially eligible patients decide to pursue kidney transplant is limited. In a recent qualitative evidence synthesis, Jones et al^5^ identified only 37 qualitative studies exploring factors influencing patient decision-making to choose or decline a kidney transplant—a relatively modest number given broad inclusion criteria and a 21-year publication timeframe. An important limitation of this review was that the included articles “did not explicitly focus on the stated phenomena of interest”, highlighting the need for research focused specifically on the decision of the patient to pursue or decline kidney transplant. Moreover, this topic has been largely unexamined in Canada. Rosenthal et al^6^ studied why individuals chose nocturnal hemodialysis over kidney transplant while Khallili et al^7^ assessed patient perspectives on the decision to accept or refuse a deceased donor kidney offer, but neither explored perspectives on kidney transplantation in general. To date, no study has broadly examined the perspectives toward kidney transplant amongst individuals with kidney failure in Canada.

Importantly, patient attitudes towards transplant are potentially modifiable and may be influenced by standardized patient information systems and personalized decision support tools enabling fully informed treatment decisions.^8^ A broad understanding of patient perspectives regarding kidney transplantation may inform education strategies and interventions to encourage and improve access to transplantation for all eligible patients with kidney failure.^9^ Therefore, the objective of this study was to examine the perspectives of individuals with kidney failure in Canada regarding kidney transplant as a potential treatment option.

## Methods

This study employed interpretive description,^10^ a flexible and pragmatic qualitative approach focused on the generation of directly applicable knowledge for use in healthcare and other applied fields. This study was approved by the Nova Scotia Health Research Ethics Board (REB file #: 1028265).

### Participants and Recruitment

Incident dialysis patients aged 18-75 years (within 6 months of starting either hemodialysis or peritoneal dialysis) or patients with an eGFR ≤14 mL/min/1.73m^2^ and within 3 months of pre-emptive kidney transplant referral, were recruited from a mix of academic and community-based dialysis centers in 4 Canadian provinces (i.e., Nova Scotia, Ontario, Manitoba, and Saskatchewan). Purposive sampling^11,12^ was used to identify individuals with knowledge and experiences directly relevant to the phenomenon of interest (i.e., consideration of kidney transplant as a treatment option) and to improve representation by age, gender, and clinical characteristics (e.g., pre-dialysis nephrology care and comorbidity burden).

Across provinces, individuals who met the study criteria were identified in the clinic setting, informed of the study by local team members, and asked if they would authorize the sharing of their contact information with the research team. Those who provided authorization were contacted by the Research Coordinator who provided additional information, confirmed participant eligibility and interest, and obtained informed consent.

### Data Collection

Consented participants completed an intake survey which captured relevant clinical and demographic information and were scheduled to participate in a virtual one-time focus group discussion (FGD). Individuals who were unable to attend a FGD due to scheduling challenges were invited to participate in an individual interview. FGDs and interviews explored patient perspectives on kidney transplantation as a treatment strategy for kidney failure and potential concerns or perceived barriers to pursuing transplant. A preliminary FGD with healthcare providers in Halifax, Nova Scotia, was conducted to inform data collection. In this FGD, providers reflected on prior conversations with patients in the clinical context. Five themes pertaining to patient decision-making regarding transplant were identified: “improved health”, “risk tolerance”, “preparedness”, “frustration”, and “guilt”. These findings were considered alongside literature from other jurisdictions, to inform the development of the FGD and interview guide (Supplementary File 1).

FGDs and interviews were conducted in English using video-conferencing technology (Microsoft Teams), which enabled concurrent participation from different locations. All FGDs included males and females to ensure an adequate number of participants in each, and to facilitate the timely conduct of FGDs following initial recruitment. Separate FGDs were held for dialysis and non-dialysis patients to allow the team member conducting the focus groups to tailor the questions and prompts to each group. FGDs and interviews were led by a team member (CK), a qualitative researcher with extensive experience conducting focus groups and interviews with various patient populations. A second team member (JI; the Research Coordinator) attended FGDs and interviews to assist with software operation and technical issues, and gain familiarity with the data to support analysis. No relationship existed between these team members and participants. All sessions were recorded and transcribed verbatim using the transcription feature in Teams with participant consent. Transcripts were verified for accuracy by the Research Coordinator prior to analysis and direct identifiers were removed.

### Data Analysis

Data were analyzed using thematic analysis as described by Braun and Clarke.^13^ This approach provides a systematic and rigorous framework for identifying patterns within qualitative data, and is primarily inductive, ensuring emergent themes reflect the perspectives and experiences of participants. Data from interviews and focus group discussions were combined for analysis, adding to the overall richness of the dataset and allowing for the triangulation of data across data sources, thereby improving the trustworthiness of the findings.^14^

To promote rigor, analyses were undertaken by two team members (the same individuals who were involved in data collection). Analysis was led by the team member with expertise in qualitative research (CK), who trained the Research Coordinator (JI) in qualitative analysis techniques (i.e., coding and theme development). At the time of this study the Research Coordinator was involved in a broader program of kidney transplant research and thereby possessed important content expertise.

The two team members first reviewed each transcript to re-familiarize themselves with the data and independently coded two transcripts. Next, they met to compare codes, discuss their conceptual basis, and identify and reconcile inconsistencies. The resulting list of codes was used to guide analysis of subsequent transcripts. Coding was done separately by each of the two team members, who met after every 2 transcripts to discuss and further refine codes as needed. Initial themes (i.e., patterns within the data) were identified by the two team members and refined through ongoing discussion with members of the larger study team (experts in nephrology and kidney transplant). As incoming data were analyzed and discussed, team members considered whether any new ideas or concepts were raised during the most recent focus group/interview conducted (e.g., new codes), whether there were any concepts or ideas that would benefit from a more in-depth examination in subsequent interviews, whether there were additional perspectives that were needed, and whether the data collected were sufficiently robust to support the emergent themes. Once the team was satisfied that informational redundancy (i.e. saturation) had been achieved, and that ongoing data collection was unlikely to provide additional insights, recruitment was stopped.

This iterative process was facilitated using qualitative data analysis software (NVivo, QSR International). This software also captured the various iterations of coding, providing an audit trail. Illustrative quotations reflecting each theme were selected to support the analysis. Direct identifiers were removed from transcripts (i.e., names of individuals, cities, institutions) and paraphrasing used to protect anonymity. Study reporting was based on the Standards for Reporting Qualitative Research (SRQR),^15^ included in Supplementary File 2.

## Results

### Participants

Fifty individuals were referred to participate in the study and 28 consented. Thirteen of the individuals who consented did not participate due to a change in eligibility status, loss of contact, or scheduling conflict. A total of 15 individuals participated in a focus group (n=13) or interview (n=2). Of those who participated, seven were on dialysis and eight were pre-dialysis (four and six of whom had been referred for transplant, respectively). Participant characteristics are summarized in Table 1.

**Table 1.** Participant Characteristics

Characteristic		n (%)
Age, years (mean ± SD)	​	62 ± 9.3
Province of residence	Manitoba	2 (13)
	Nova Scotia	9 (60)
	Ontario	1 (7)
	Saskatchewan	3 (20)
Biological sex	Male	8 (53)
	Female	7 (47)
Gender	Man	8 (53)
	Woman	7 (47)
Ethnicity	Caucasian	13 (87)
	Other	2 (13)
Dialysis status	On dialysis	7 (47)
	Hemodialysis	5 (33)
	Peritoneal dialysis	2 (13)
	Pre-dialysis	8 (53)
Transplant status	Referred	10 (67)
	Activated	4 (27)
	Living donor work-up within 1 year of referral	4 (27)
Cause of kidney failure	Diabetes	4 (27)
	Polycystic kidney disease	4 (27)
	IgA nephropathy	2 (13)
	Other	5 (33)
Comorbidities^*^, number	1	9 (60)
	2	3 (20)
	3	2 (13)
	4+	1 (7)

Abbreviations: SD=standard deviation.

^*^Participants selected “all that apply” from the following list: diabetes, cerebrovascular disease, history of prior malignancy, prior failed kidney transplant, history of depression, other mental health diagnoses, coronary artery disease, congestive health failure, peripheral vascular disease, hypertension, atrial fibrillation, liver disease.

### Themes

**Figure 1.**
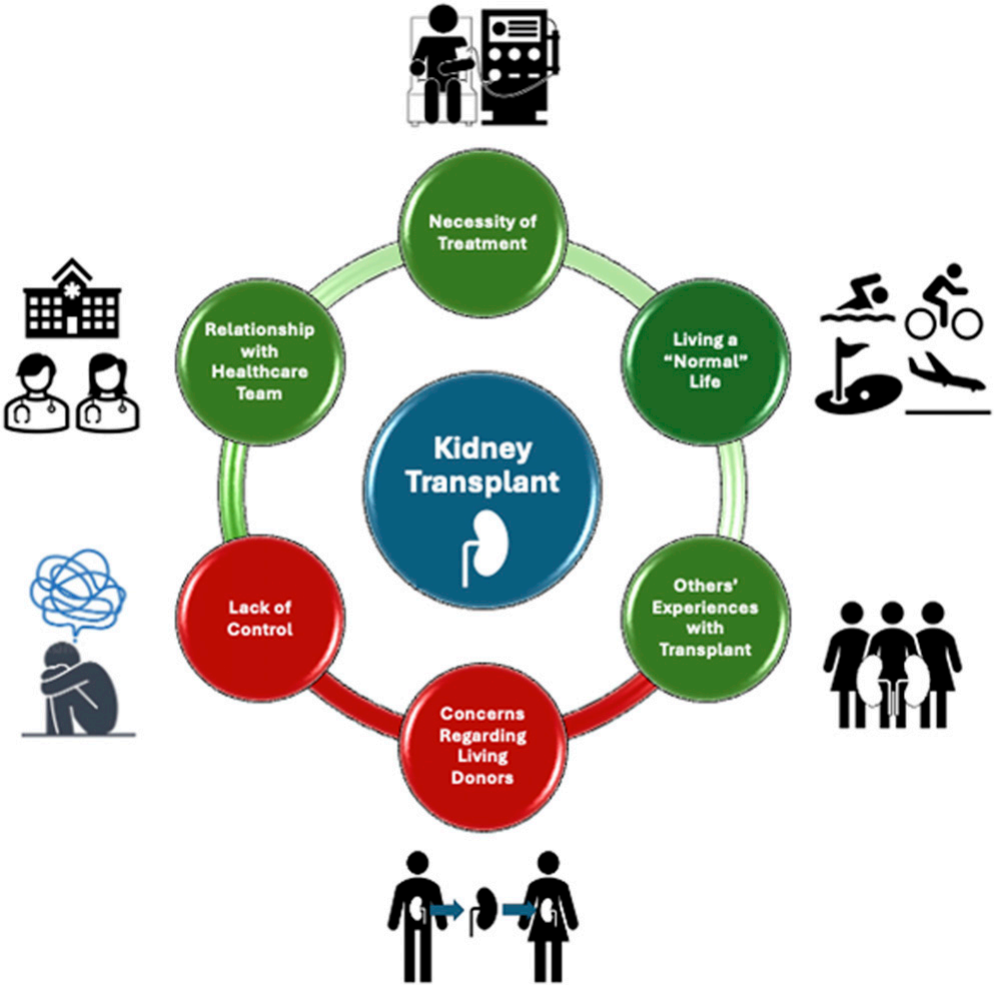
Summary of major themes regarding patient perspectives towards kidney transplantation

Six themes were identified. Overall, participants expressed a willingness to consider transplant, and a clear preference for transplant compared to dialysis. Participants’ willingness to consider transplant was influenced by their (1) *relationship with the healthcare team*, recognition of (2) *the necessity of treatment*, the (3) *potential to live a “normal” life*, and knowledge of (4) *others’ experiences with transplant*. Despite a preference for transplant, participants expressed (5) *concerns regarding securing a living donor* and a sense of having (6) *a lack of control* over the transplant referral process. Themes are described below and summarized in Figure 1. Illustrative quotes are provided in Table 2.

**Table 2.** Illustrative Quotes

Theme	Illustrative quotes
Relationship with the healthcare team	*“*I mean, I’ve talked to a lot of the doctors, the kidney doctors, the transplant doctors, and they are very, very professional and they’re very, very knowledgeable. And any questions I’ve had, they’ve been able to answer them, so I really don’t have the concerns. You know, I think that certainly in [city] at the kidney clinic, it is a clinic that’s got, like I said, all sorts of different professionals in it. They seem to have a very disciplined approach to it and stuff like that. And they’ve done it very, you know, quite a few times, though, I really don’t have any concerns.*” *[P1]
	*“*And I think that’s probably my biggest issue is that I just wasn’t informed about that. Like had I like been given the information…, I might have not agreed to that [blood transfusion], which I could have, you know, said,* '*no, I don’t want it*'. *But I didn’t know that. I didn’t know that would [cause antibody formation], which took my chances of getting a transplant from like six months to a few years to possibly 10 years now. Which kind of really disappointed me.*” *[P2]
Necessity of treatment	*“*I don’t have a lot of positive thoughts about dialysis other than I’m gonna try to make it as much as a positive experience for myself as I can. I’d like to do it in the evening so I can still remain active during the day. Things like that. I see it as that’s necessary to sustain your life, right?*” *[P3]
	*“*I mean, obviously surgery is surgery, right? I mean, you know, nobody likes to have to go to surgery, but I mean, in the end the outcome if you don’t [have it done] is probably a lot worse than having it done, right?* ” *[P1]
	*“*The only thing I have is that as long as I wake up [from surgery], that’s my concern. I’m a little blunt. I apologize, but that’s my thinking. I mean, that’s it. I gotta do this.*” *[P4]
Living a “normal” life	*“*So the transplant for me, I think to do that would have let me go back to work. And also like, go on vacation and you know, go places. My husband and I are both getting to that age where I mean in five to seven years we’re probably both thinking of retiring anyway [.] And we had, you know, planned, like we had bought this place [location] to be able to go to it in the winter time and in the summer time. And uh, that was our plan and maybe do some traveling which on dialysis isn’t gonna happen, but with the transplant at least [we could] travel within Canada at least.*” *[P2]
	*“*Uh, basically [I chose home dialysis] so I could still go to work. I can do the dialysis at night while I’m sorta, while I’m sleeping, half the time it keeps me awake. Uh, because basically, that’s so I could stay working during the day and I can do all that stuff at night and keep somewhat used to the way I used to be.*” *[P5]
	*“*And then yeah, and you know, because I’m still at the age, right, I still need to work. And work to me is like a privilege. It is a blessing. That’s how I see work. So, I think [getting a transplant] is going to be very good for my mental health to be able to work well if it is more efficiently without any of these [dialysis] tubes in my tummy.*” *[P6]
Others’ experiences with transplant	*“*Well, again, like going from my sister, OK? She was pretty close to her deathbed to, you know, living now for [many years] without really any issues. Yeah, the medicine I guess would be a pain that you gotta take it at the same time every day. But even that over the years for her has gone from, you know, and I don’t know exactly the numbers, but we’ll say from 10 pills down to two pills every single morning at 9:00 AM…She goes out. She travels. The whole kaboodle. Where again, you know, she was pretty close to her deathbed otherwise.*” *[P7]
	*“*I’ve had improved feeling with my health, my health condition, since they started peritoneal dialysis, but I think it would be better with transplant. I can say that because [my family member had a kidney transplant] and before that transplant, he was like, he was so unwell and he was a sportsman. But a few weeks after the transplant, he was back to* [being active]*.*” *[P6]
	*“*I feel very positively about transplantation or transplants because my family members who have had it have been able to live a good lifestyle. Yes, there’s a lot of medications involved in it, and immunosuppressants and stuff, and side effects, but I see that as more positive than dialysis.*” *[P3]
Concerns with living donors	*“*My wife wanted to try to donate one to me and my kids are trying to […]. But I’d feel like crap if something happened to them because they only had one kidney to deal with, so that part would bother me.*” *[P5]
	*“*My sons donating, you know, I have a bit of an issue with that. Umm, because you know it’s something you don’t want your sons to have to go through type thing, but they’ve offered and gone through the, you know, the process or started the process on that, and if it’s a perfect match then that’s fine. If it’s not a perfect match, I might suggest that they don’t go through it, but we’ll see.*” *[P1]
	*“*I haven’t pushed the idea of searching for a personal donor. I find that that’s a hard place for me to go right now, to ask somebody to, because my fear would be that something would happen to that person if they gave me a kidney and then all of a sudden they got sick or something happened to them. I don’t know if I could. Yeah, that’s the part I’m trying to get over. Get over that hump. Sort of like to feel comfortable with going to ask for some of your friends or family or, uh, for donation.*” *[P8]
	*“*I used to live in [province] and they said, well, you know, what about your children? I said I didn’t have children so I could take parts from them. That was not something I could do. So, you know, and the thing that concerns me is I, I don’t know why I got kidney disease in the first place. So what if they develop it later, and then they only have one kidney, right?*” *[P9]
Lack of control	*“*One of my donors, who was super willing, she came, had all the tests done that were required initially, and she was informed that we were not matched and she was allowed to think of donating [through an organ donation exchange program]…That part at first I did not like, but you know, as I said, who can? There’s some things beyond our control, that was in their decision in their conversation.*” *[P6]
	*“*No, [the living donor healthcare team], they’re waiting. They won’t do it. Which I think is kind of stupid. I mean, that’s just my opinion. Like I don’t know what all their protocols are, and I definitely don’t understand some of them so. You know, I don’t make the rules. So it’s a little frustrating that way when you have somebody who’s willing and able and available and they won’t proceed. Like I can’t see why they can’t do the testing and have that all done and still work on getting the BMI down.*” *[P10]
	*“*…my one sister has to lose a lot of weight, and she is working hard at that but has to lose a lot of weight to even be tested [to see if she is a match]. They won’t test her. And my other sister unfortunately [required treatment for another health issue which delayed testing] and it’s just been, I don’t know, like, stressful. And I think that, like, all they had to do was do a blood test to, you know, there’s a good chance that they’re not going to match.* ” *[P2]

#### (1) Relationship With the Healthcare Team

Healthcare teams play an important role in how patients regard transplant, and their overall care experience. Most participants in this study described having positive relationships and experiences with members of their nephrology team (i.e., nephrologists, transplant coordinators, nurses) and felt that treatment recommendations were made with their best interests in mind. This trust in their healthcare team— in the teams’ knowledge, skills, and beneficence— contributed to participants’ willingness to undergo transplant and helped mitigate concerns related to the surgery itself. At the same time, the need for improved communication was cited by several participants. Specifically, about what to expect regarding the overall transplant process (i.e., what needs to happen between deciding to pursue transplant and getting a transplant), recovery from transplant surgery, and donor eligibility (i.e., criteria and required tests). Poor communication negatively impacted the patient experience, leading to feelings of frustration, and in some cases had direct implications for care. For example, one patient reported not being informed that their decision to receive a semi-elective blood transfusion could result in sensitization thereby delaying their transplant by several years.

#### (2) Treatment as a Necessity (“Doing What Needs to Be Done”)

Patients identified many challenges and disadvantages associated with both dialysis and transplant. Dialysis, whether done at hospital or at home, was regarded as time-consuming, inconvenient, and disruptive to everyday life. Patients reported being unable to work due to needing dialysis several days each week, being unable to travel, and having less time to spend with family and friends. These issues were compounded for patients who were required to travel longer distances to a dialysis unit (e.g. patients residing in rural areas). While home dialysis therapies mitigate the need to travel for treatment, participants felt home dialysis presented other challenges, including limited assistance from medical professionals, and costly home upgrades (e.g., to address water quality and/or electrical issues). Transplant-related challenges included the risk of surgical complications, lengthy recovery, and the need for ongoing medication (i.e., immunosuppressants). Despite these challenges and disadvantages, treatment (whether dialysis or transplant) was regarded as something that was necessary to avoid disease progression and death. As such, participants were willing to avail themselves of whatever options were available to them.

#### (3) Living a “Normal” Life

Participants’ treatment attitudes and preferences were largely informed by their desire to live a “normal” life. Although participants were grateful for dialysis, it was generally regarded as a short-term, stop-gap measure until transplant was an option. Patients looked forward to a time in the future when they would no longer be tethered to equipment for treatment and could enjoy activities that they could not enjoy while on dialysis (e.g., working full time, swimming, travelling, spending time at the cottage). Patients who chose to receive at-home dialysis did so because it was less disruptive to everyday life compared to hospital-based dialysis (e.g., less travel, less time away from family, able to do at night, etc.). Transplant, however, was seen as the end-goal. Participants perceived life post-transplant as a return to “normal”, where they would be able to do the things that they were otherwise unable to do, either because they did not feel well enough or felt constrained due to dialysis.

#### (4) Others’ Experiences With Transplant

The broad support of and preference for transplant among study participants was partly related to their knowledge of others’ transplant experiences and outcomes. Nearly all patients who participated in this study knew someone, or knew *of* someone, who had undergone transplant (kidney or other). Given the potential familial nature of some kidney disease, many of the patients in this study had other family members with kidney failure who had received dialysis and/or transplant. Participants described positive changes in the health status and quality of life of these individuals after receiving transplant, leading to positive perceptions of transplant (i.e. as a way of returning to a more “normal” life) and helping to mitigate any fears or concerns about transplant surgery.

#### (5) Concerns Regarding Securing a Living Donor

All participants indicated that they would be willing to accept a kidney from a deceased donor. When asked whether they would accept a kidney from a living donor, participant responses indicated that the decision was less straightforward. While some participants acknowledged an organ from a living donor may be preferable (i.e., due to the health of the organ), most expressed concern over the potential consequences for living donors, such as the potential for surgical complications and the risks of the donor being left with only one kidney (“what if that one fails?”). Related to this, participants expressed feelings of discomfort with the idea of asking someone for a kidney, and guilt around receiving a kidney from someone they know. In some instances, knowing the potential donor and their lifestyle habits (e.g., diet, alcohol consumption) raised concerns about whether the kidney was “good enough”. More commonly, individuals felt it was “too much” to ask someone to donate a kidney to them and did not want to put anyone in an uncomfortable position by asking. These concerns were especially pronounced when it came to receiving a kidney from a family member, and especially one’s children. Patients emphasized the importance of “matching” between the donor and recipient, and that a “perfectly matched” kidney may influence their decision to accept a living donor organ. With that said, several participants acknowledged that if a family member—including their children—offered to donate their kidney to them of their own free will and with an understanding of what was involved, they would not be in a position to refuse due to their worsening health and the uncertainty around whether and when a kidney from a deceased donor would become available.

#### (6) Lack of Control

While patients may view transplant as the most desirable treatment pathway, they felt they ultimately have very little control over the process, including whether they are worked up for transplant, whether they will receive a transplant, and if so, how long they will have to wait. There are many factors that are beyond the patients’ control, such as local practice patterns, health system capacity, organ availability, and the eligibility of potential living donors. Participants often discussed having to wait—for example, having to wait for a living donor to be found, or for an organ to become available from a deceased donor, or having to wait on tests and procedures to determine whether the participant and/or their donor were eligible. No matter the participant’s level of readiness or the strength of their desire to receive a transplant, there were many factors that were beyond their control that had to align for transplant to occur. As such, patients were left feeling in limbo, with little insight regarding what to expect in the future.

## Discussion

The decision to pursue a kidney transplant is not straightforward for many individuals with kidney failure, despite its benefits. Myriad factors have been identified as potentially deterring individuals from pursuing transplant, including; consideration of other commitments (e.g., child rearing, educational pursuits), limited access to care (e.g., rurality, medical insurance coverage), financial impacts (e.g., lost income), the social aspects of dialysis, religious beliefs, and individual perspectives and attitudes toward transplant (e.g., fear of surgery, concerns regarding medication burden).^5^ Additionally, individuals with kidney failure who have received a kidney transplant have reported various post-transplant challenges, including adherence to immunosuppressant medication, maintenance of a healthy lifestyle, as well as experiencing ongoing feelings of worry, anxiety, and guilt.^2,3^ The findings of a recent systematic review and meta-analysis, reporting that globally only 57% of patients with kidney failure were willing to undergo transplant,^16^ suggests there are variations in treatment preferences across jurisdictions.

In the current study of individuals with kidney failure in Canada, participant attitudes toward kidney transplantation were positive, with all individuals expressing a clear desire to undergo transplant. Consistent with the perspectives of individuals with chronic kidney disease published elsewhere,^5,17^ participants’ positive views of transplant, and the preference for transplant over dialysis, was primarily driven by the belief that transplant would offer a chance to live a longer and more “normal” life, providing the freedom to pursue things like travel, returning to work, and spending time with family. While the disadvantages and risks of undergoing transplant surgery were acknowledged, they did not act as a deterrent to pursuing transplant. This suggests that when the perceived risks and benefits of transplant and dialysis were compared, transplant emerged as the more favorable option. Moreover, the perceived benefits of transplant, in terms of improved health and longevity, were of such magnitude that the decision to pursue it was self-evident—transplant was regarded as the only way “back to normal”.^17^ Similar risk-benefit assessments have been demonstrated for other treatment-related decision-making processes relevant to treatment for kidney failure.^6,7,18^ This underscores the need for robust patient education and access to informational resources to support patients in accurately assessing the benefits and risks of transplant, including information about individuals’ own risk profile based on individual-level factors (e.g., comorbidity, health behaviors, etc.), and to minimize susceptibility to misinformation.^5^

While participants were quite clear on whether they would pursue transplant, the decision of whether to accept a kidney from a living donor was more complex. While some research has indicated a preference for a living donor,^5,19^ participants in this study seemed to prefer, or at least be more comfortable with, deceased donation. When considering live donor transplant, prior studies have suggested an unwillingness amongst many patients to burden loved ones, fears regarding the donor’s health, and feelings of guilt as reasons for declining a living donor transplant.^23 24^ Likewise, in the current study, many participants had concerns over the health implications for living donors, and related to this, expressed discomfort with seeking a living donor. This aligns with prior research, which reported greater levels of guilt experienced by living donor recipients compared to deceased donor recipients.^3,20,21^ At the same time, many participants in this study acknowledged that, due to their poor and/or deteriorating health, they were not in a position to refuse any kidney that was *offered* to them. These findings are similar to those reported by Martin et al^19^ who examined kidney donation preferences among patients waiting for transplant in New Zealand. Patients in that study also expressed high levels of concern over donor wellbeing post-transplant yet indicated that they would accept a kidney from a living donor, with many participants commenting that they would ultimately accept any organ that they were offered. Thus, patients with kidney failure may experience less moral guilt related to the health implications for donors when an offer of donation is made compared to when they are in the position of asking. It is also possible that the preference for deceased donors in this study was related to participants’ dialysis experience. For example, individuals who have been on dialysis for an extended length of time may be more motivated to search for a living donor to expedite the transplant process.^5,7,19^ Participants in this study had been on dialysis for a relatively short period of time (no more than six months) or not at all. As such, they may not have reached a point where they were motivated to actively seek a kidney from a living donor. Given the gap between the number of individuals on the kidney transplant waitlist, and the limited number of available kidneys,^22^ timely access to transplant may be dependent on the identification of a living donor. Individuals with kidney failure may be more empowered to actively pursue living kidney donation as an option if they have an improved understanding of the actual risks to living donors—which are considered minimal^23^— and how to initiate conversations regarding living kidney donation. Notably, several patients commented on a good “matched” kidney influencing their decision to accept a living donor organ. This highlights an area for improved education, given that even a poorly human leukocyte antigen (HLA) matched living donor kidney typically yields excellent post-transplant outcomes. Concerns regarding blood group or HLA incompatibility (on account of pre-formed donor-specific antibodies) can be addressed through paired kidney donor exchange programs and should not dissuade living donor transplant (particularly in the setting of highly sensitized candidates who may also have difficulty receiving a deceased donor organ).

Importantly, the findings of this study also indicate that the experience of individuals with kidney failure is characterized by feelings of uncertainty and a lack of control. This was evident in various comments about not knowing what the transplant process involved, patients not knowing where they were in terms of the process (i.e., which tests needed to be done), and uncertainly around how long it would take for a kidney to become available, or if one would become available at all. This highlights the need for improved communication between the transplant team and the patient, which was explicitly identified by participants in this study as an aspect of care that should be improved and widely reported elsewhere as a gap to be addressed.^5,24^ These findings also reflect the uncertainty that is inherent to organ transplant, especially transplant involving a deceased donor. In Canada, wait times for a deceased donor kidney offer are unpredictable and can be lengthy (average of nearly 4 years) with a proportion of individuals dying while on the waitlist due to a shortage of available organs.^22^ When a deceased donor kidney does become available, the decision of whether or not to accept it is made by the transplant nephrologist and transplant surgeon at the provincial or regional transplant program where the patient is waitlisted.^7^ This process is largely out of patients’ hands and cannot be expedited unless a living donor is identified. Patients may struggle with navigating indefinite wait times and the feeling that they are “living in limbo” without a clear path forward.^17^ This uncertainty may have negative implications for patients’ psychosocial wellbeing.^25^ Patients with kidney failure may therefore benefit from resources and tools to prepare them for the possibility of a lengthy wait for transplant, as well as access to ongoing emotional and psychosocial support.

Although gaining attention, qualitative studies remain relatively uncommon in transplant literature despite their tremendous value and the increasing emphasis on patient-centeredness in research and decision-making.^26,27^ For example, qualitative studies have been used to inform clinical care and policy development relevant to kidney failure, as well as the development of guidelines to support shared decision-making relevant to treatment.^27^ However, overall perspectives toward transplant as a management strategy for kidney failure remains largely unexplored, and never in a Canadian context. While this study has important strengths including being the first to broadly examine perspectives towards kidney transplantation amongst individuals with kidney failure in Canada, there are several limitations. Firstly, this study included a relatively small sample and may not be representative of the broader population of individuals with kidney failure in Canada. Regardless, the sample size was sufficient to achieve thematic saturation, and it was not felt a larger sample size would further enrich the data. Secondly, despite attempts to recruit participants from a range of backgrounds, racially diverse, LGBTQ2S+, and other equity-deserving groups were under-represented in this study. Related to this, study findings may have differed if there was greater representation from communities with a historical mistrust of the healthcare system and public institutions more broadly. Thirdly, the conversations that occurred during the FGDs were somewhat hypothetical and did not necessarily reflect how decision-making would unfold in a clinical encounter under the advice of a clinician. Additionally, patient partners were not involved in the development of the FGD and interview guide. Finally, participants had already predominantly been referred for transplant at the time of study consent, resulting in a potentially biased study population. The majority of study participants would have had conversations with their clinical team about transplantation and had their questions and concerns addressed. This may have resulted in a study population that was more informed and motivated to pursue transplantation, compared to a population including those who had not yet been referred, or who had declined referral. It has also been shown that patients with greater health literacy and those reporting better patient-clinician relationships are more likely to participate in research activities.^28,29^ Although we did not directly assess health literacy, the findings of the study may have been influenced by self-selection bias and may not be representative of the broader population of interest. Nonetheless, given the paucity of Canadian research on this topic, this study represents a novel contribution to the literature and provides several key insights into treatment related decision-making by individuals with kidney failure in Canada.

## Conclusion

Understanding patient perspectives regarding kidney transplantation in the pre-transplant context provides important insights into decision-making processes, and how these can be best supported to improve access to kidney transplant for individuals with kidney failure. Although all participants in this study were willing to undergo transplant and to accept a kidney from a living donor if offered, concerns were raised regarding seeking a living kidney donor and feelings regarding loss of control throughout the process. The findings of this study highlight several issues that may undermine transplant uptake and negatively impact quality of life post-transplant, including informational gaps, feelings of uncertainty, and moral guilt. These findings also point to the need for improved information, education, communication and psychosocial supports to empower patients to confidently make treatment-related decisions that are evidence-informed and aligned with individual needs, preferences, and values. Additional research is needed to examine a broader range of perspectives on kidney transplantation, including the perspectives of individuals who have not yet been referred for or who have declined transplant, and the perspectives of racially diverse, LGBTQ2S+, and other equity-deserving groups.

## Supplemental Material

Supplemental material - Perspectives Toward Kidney Transplantation Amongst Individuals with Kidney Failure in Canada: A Qualitative Study Supplemental material for Perspectives Toward Kidney Transplantation Amongst Individuals with Kidney Failure in Canada: A Qualitative Study by Cynthia Kendell, Jad Issa, Bethany Foster, Karthik Tennankore, and Amanda J. Vinson in Canadian Journal of Kidney Health and Disease.

Supplemental material - Perspectives Toward Kidney Transplantation Amongst Individuals with Kidney Failure in Canada: A Qualitative Study Supplemental material for Perspectives Toward Kidney Transplantation Amongst Individuals with Kidney Failure in Canada: A Qualitative Study by Cynthia Kendell, Jad Issa, Bethany Foster, Karthik Tennankore, and Amanda J. Vinson in Canadian Journal of Kidney Health and Disease.

Supplemental material - Perspectives Toward Kidney Transplantation Amongst Individuals with Kidney Failure in Canada: A Qualitative Study Supplemental material for Perspectives Toward Kidney Transplantation Amongst Individuals with Kidney Failure in Canada: A Qualitative Study by Cynthia Kendell, Jad Issa, Bethany Foster, Karthik Tennankore, and Amanda J. Vinson in Canadian Journal of Kidney Health and Disease.

## Data Availability

The data collected for this study are not publicly available.*
