# Supplementary material for: Perspectives Toward Kidney Transplantation Amongst Individuals With Kidney Failure in Canada: A Qualitative Study
Source: Can J Kidney Health Dis. 2026 Aug 12;13:20543581261472120. doi: 10.1177/20543581261472120 (PMC13469535; doi:10.1177/20543581261472120)
Supplement: Supplementary file 2 [file sj-pdf-2-cjk-10.1177_20543581261472120.pdf]

APPENDIX B. SRQR reporting checklist

	Item Description	Location (or reason for not reporting)
**Title & Abstract**		
Title	Describe the nature and topic of the study. Identify the study as qualitative or indicate the approach or data collection methods.	First page
Abstract	Summarise the key elements of the study using the abstract format of the intended publication.	First page
**Introduction**		
Problem Formulation	Describe the problem/phenomenon studied, its significance, relevant theory and empirical work, and gaps in current knowledge.	Introduction, paragraphs 1-3
Purpose or research question	Describe the purpose of the study and specific objectives or questions.	Introduction, paragraph 3
**Methods**		
Qualitative approach and research paradigm	Describe your qualitative approach, your guiding theory (if appropriate), and research paradigm, and reasons for your choices.	Methods, paragraph 1
Researcher characteristics and reflexivity	Describe how researchers' characteristics may influence the research, including personal attributes, qualifications/experience, relationship with participants, assumptions, and/or presuppositions; potential or actual interaction between researchers' characteristics and the research questions, approach, methods, results and/or transferability.	Methods, Data Collection, paragraph 2<br><br>Methods, Data Analysis, paragraphs 2 and 3
Context	Describe the setting/site(s) in which the study was conducted, why it was selected, and any other salient contextual factors that may influence the study.	Methods, Participants and Recruitment, paragraph 2.
Sampling strategy	Describe how and why research participants, documents, or events were selected; criteria for deciding when no further sampling was necessary, and the rationale for those criteria.	Methods, Participants and Recruitment, paragraphs 1-2
Ethical issues pertaining to human subjects	Describe any approval by an appropriate ethics review board and participant consent, or explain any lack thereof. Describe any other confidentiality and data security issues.	Methods, paragraph 1 (ethics)<br><br>Methods, Data analysis, paragraph 2 (de-identification of data)
Data collection methods	Describe the types of data collected; details of data collection procedures including (as appropriate) start and stop dates of data collection and analysis, iterative process, triangulation of sources/methods,	Data Collection, paragraphs 1-2 (REB approval information)

	and modification of procedures in response to evolving study findings. Describe your rationale for these choices.	
Data collection instruments and technologies	Describe any instruments (e.g., interview guides, questionnaires) and devices (e.g., audio recorders) used for data collection; describe if/how the instrument(s) changed over the course of the study.	Appendix A (focus group/interview guide).
Units of study	Describe the number and relevant characteristics of participants, documents, or events included in the study. Describe the level of participation.	Results, Participants, paragraph 1 (participant characteristics)<br><br>Methods, Data Collection, paragraph 1-2 (level of participation)
Data processing	Describe the methods for processing data prior to and during analysis, including transcription, data entry, data management and security, verification of data integrity, data coding, and anonymisation / deidentification of excerpts.	Methods, Data Collection, paragraph 1-2 (for details re: verification, data management and security)<br><br>Methods, Data Analysis,
Data analysis	Describe the process by which inferences, themes, etc. were identified and developed, including the researchers involved in data analysis; usually references a specific paradigm or approach. Describe why you chose this process.	Methods, Data Analysis, paragraph 1-2
Techniques to enhance trustworthiness	Describe any techniques to enhance trustworthiness and credibility of data analysis,(e.g., member checking, triangulation, audit trail). Describe why you chose these techniques.	Methods, Analysis, paragraph 2 (double coding, audit trail, discussion with broader team)
**Results**		
Synthesis and interpretation	Describe the main findings (e.g., interpretations, inferences, and themes); might include development of a theory or model, or integration with prior research or theory.	Results, Themes, paragraphs 1-7
Links to empirical data	Provide evidence (e.g., quotes, field notes, text excerpts, photographs) to substantiate analytic findings.	Table 1 (illustrative quotes)
**Discussion**		
Integration with prior work, implications, transferability, and contribution(s) to the field	Summarize the main findings, explain how findings and conclusions connect to, support, elaborate on, or challenge conclusions of earlier scholarship; discuss the scope of application/generalizability; identify unique contribution(s) to scholarship in a discipline or field.	Discussion, paragraphs 1-5

Limitations	Discuss the trustworthiness and limitations of findings	Discussion, paragraph 5
**Other**		
Conflicts of interest	Describe any potential sources of influence or perceived influence on study conduct and conclusions. Describe how these were managed.	Not applicable (no conflicts of interest)
Funding	Describe sources of funding and other support. Describe the role of funders in data collection, interpretation, and reporting.	Acknowledgements
