# Supplementary material for: Perspectives Toward Kidney Transplantation Amongst Individuals With Kidney Failure in Canada: A Qualitative Study
Source: Can J Kidney Health Dis. 2026 Aug 12;13:20543581261472120. doi: 10.1177/20543581261472120 (PMC13469535; doi:10.1177/20543581261472120)
Supplement: Supplementary file 3 [file sj-pdf-3-cjk-10.1177_20543581261472120.pdf]

CJKHD-26-0025.R1

Perspectives Toward Kidney Transplantation Amongst Individuals with Kidney Failure in Canada: A Qualitative Study

RÉSUMÉ

Introduction : La compréhension des perceptions de la transplantation rénale chez les personnes atteintes d'insuffisance rénale est essentielle à la prise de décision thérapeutique et à la préparation à la vie après la transplantation, mais cette question n'a pas encore été étudiée dans le contexte canadien. Cette étude visait donc à examiner les perceptions de cette population de patients à l'égard de la transplantation rénale.

Méthodologie : Étude sous forme de groupes de discussion et d'entrevues individuelles menée auprès d'adultes (18 à 75 ans) issus de quatre provinces canadiennes et ayant amorcé la dialyse au cours des 6 mois précédents ou ayant été orientés de façon préventive pour une transplantation rénale au cours des 3 mois précédents. Les données ont été analysées à l'aide d'une analyse thématique inductive.

Résultats : En tout, 15 personnes ont participé à l'un des quatre groupes de discussion (n=13) ou à une entrevue semi-structurée (n=2), soit 7 personnes sous dialyse et 8 au stade prédialyse, desquelles respectivement 4 et 6 personnes avaient été orientées pour une transplantation rénale. Dans l'ensemble, les participants ont exprimé une nette préférence pour la greffe par rapport à la dialyse. Quatre facteurs influençaient la volonté d'envisager une transplantation rénale : (1) *la relation avec l'équipe soignante*, (2) *la reconnaissance de la nécessité du traitement*, (3) *la possibilité de retrouver une « vie normale »* et (4) *la connaissance des expériences d'autres personnes transplantées*. Malgré une préférence pour la transplantation, les participants ont

exprimé (5) *des préoccupations quant à la possibilité de trouver un donneur vivant* et (6) *un sentiment de perte de contrôle* sur le processus d'orientation vers la transplantation.

Conclusion : Cette étude évaluait les perceptions de la transplantation rénale chez des personnes atteintes d'insuffisance rénale au Canada. Une meilleure compréhension des préoccupations des patients à l'égard d'une éventuelle transplantation pourrait favoriser l'élaboration de stratégies éducatives, communicationnelles et psychosociales mieux adaptées et renforcer la capacité des patients à prendre des décisions thérapeutiques avec confiance.
